# Early‐life temperature drives recruitment success in Eurasian perch ( *P* *erca fluviatilis*) populations

**DOI:** 10.1111/jfb.70541

**Published:** 2026-06-28

**Authors:** Valentin Cavoy, Jean Guillard, Cécilia Barouillet, Orlane Anneville, Najwa Sharaf, Christian Gillet, Chloé Goulon

**Affiliations:** ^1^ University Savoie Mont Blanc, INRAE, CARRTEL Thonon‐les‐Bains France; ^2^ OFB, INRAE, University Savoie Mont Blanc, Pôle R&D ECLA France; ^3^ Aix Marseille Univ., INRAE, RECOVER Aix‐en‐Provence France

**Keywords:** climate change, embryonic development, lake ecosystems, statistical modelling, temperature effects

## Abstract

Interannual fluctuations in the abundance of young‐of‐the‐year (YOY) European perch (*Perca fluviatilis*) were studied in two large French peri‐alpine lakes using a 12‐year dataset of late summer hydroacoustic surveys. Previous research has highlighted the importance of temperature. However, these studies often considered only monthly or seasonal averages. We analysed 12 years of data at the ecosystem level to identify the drivers of annual fluctuations in YOY perch stocks. We used traditional thermal indices based on average values, as well as thermal indices reflecting daily temperature fluctuations, derived from 36 years of in situ perch spawning data. Embryonic thermal conditions alone accounted for between 44% and 88% of the variation in YOY perch density across the lakes. An increase in temperature during the embryonic phase was beneficial, whereas significant daily variations had the opposite effect. In contrast, trophic factors and the adult stock did not exhibit consistent trends. Our study confirms previous findings using ecosystem‐scale analyses, long‐term data and novel thermal indices, validating in natura the central role of spring thermal regimes in shaping recruitment and improving our understanding of recruitment variability under climate change.

## 1 INTRODUCTION

Interannual variability in fish recruitment remains one of the most enduring challenges in ecology (Houde, 2008; Takasuka et al., 2021). In both marine and freshwater systems, explaining and predicting year‐class strength is difficult because of the complex interplay between environmental and biological drivers (Brucet et al., 2013; Houde, 2008; Marcek et al., 2021). Despite decades of research, several influential conceptual frameworks, including the critical period (Hjort, 1914), match–mismatch (Cushing, 1990) and larval size hypotheses (Miller et al., 1988), have not yielded consistent predictive power across systems. However, a common thread among these hypotheses is temperature‐driven processes. Temperature governs nearly every stage of early development in fishes. It controls embryogenesis, hatching timing, yolk absorption, larval growth and metabolism (Ayala et al., 2015; Ojanguren & Braña, 2003). It also determines the duration and onset of the transition from endogenous to exogenous feeding, a critical bottleneck in recruitment, particularly in clupeid species such as herring (Houde, 2008).

Temperature can also indirectly affect fish recruitment. The match–mismatch hypothesis, for instance, is ultimately temperature‐dependent: larval hatching must align with the phenology of zooplankton growth, which is governed by thermal cues (Cushing, 1990; Peck et al., 2012). Similarly, the critical period hypothesis hinges on the pace of developmental progression and the metabolic demands imposed by temperature (Ayala et al., 2015; Ojanguren & Braña, 2003). Together, these frameworks converge on the idea that temperature is not merely one variable among others but shapes the timing, success and synchrony of early‐life‐stage processes (Laurel et al., 2021).

For Eurasian perch (*Perca fluviatilis*), early development occurs during a short and thermally sensitive spring window (Craig, 1987; Gillet & Dubois, 1995, 2007) and remains highly sensitive to thermal conditions. The direct effect of temperature during early life stages on the recruitment of both *P. fluviatilis* and *P. flavescens*, two species that are morphologically and ecologically similar (Thorpe, 2011), has been investigated since the 1980s (Fraz et al., 2024, 2025; Guma'A, 1978; Linløkken, 2023; Ning et al., 2025; Schmitz & Sepulveda Villet, 2021; Wang & Eckmann, 1994). For example, a wind‐induced decrease in surface water temperatures in spring can delay warming trends and cause high mortality (Clady, 1976; Van De Hey et al., 2013).

However, temperature is not the only factor affecting early perch development (Craig, 2000). Wind can act as a mechanical stressor by physically disrupting the egg ribbons laid in shallow areas, displacing or damaging them and reducing hatching success (Clady & Hutchinson, 1975). Biotic factors such as zooplankton density, spawner abundance and cannibalism add further complexity to recruitment dynamics (Houde, 2008). Zooplankton availability is particularly critical for larval perch, which are selective feeders that require appropriately sized prey during a narrow post‐hatching window (Wang, 1994). Moreover, adult perch density can have both positive effects through increased reproductive output and negative effects via intraspecific predation and competition among perch juveniles (Craig, 2000; Dubois et al., 2008; Kokkonen et al., 2019).

Previous studies have mostly assessed the effects of temperature under controlled conditions, typically in experimental systems at constant temperatures (Fraz et al., 2024, 2025; Guma'A, 1978; Van De Hey et al., 2013). Although other studies have been conducted on natural ecosystems, they have relied on point‐in‐time indicators of perch density, such as bottom trawl surveys, gillnets and traps, which are not representative of the entire ecosystem, or on commercial fishing landings, which are known to be biased (Clady, 1976; Clady & Hutchinson, 1975; Honsey et al., 2016). Furthermore, these studies often compare young‐of‐the‐year (YOY) density or aquarium survival to broad‐spectrum indicators such as the mean spring–summer temperature (Clady, 1976; Honsey et al., 2016). Few studies have validated these findings using long‐term ecosystem‐scale monitoring. Likewise, few have used high‐resolution thermal indices capable of capturing short‐term temperature fluctuations during early developmental stages, despite growing evidence that temperature variability can strongly influence biological responses (Vasseur et al., 2014).

In this study, we assessed the influence of key environmental and biological drivers, focusing primarily on temperature as a stage‐specific, phenology‐aligned predictor of recruitment in Eurasian perch. Daily temperature fluctuations during incubation and larval development result in a complex thermal profile. To resolve this, we tested several thermal indices, each capturing different aspects of the thermal regime, to identify the one that best explains YOY perch density. We hypothesized that compared with conventional seasonal temperature means, dynamic temperature metrics, which are based on the biological timing of early development, are stronger predictors of YOY perch abundance. To test this hypothesis, we used YOY density proxies derived from hydroacoustic surveys conducted between 2011 and 2023, combined with environmental and biological monitoring in two deep French peri‐alpine lakes, Annecy and Bourget (Rimet et al., 2020). Recruitment variability was then related to indices describing water temperature, wind speed, zooplankton availability and adult perch abundance.

## 2 MATERIALS AND METHODS

### 2.1 Study sites

Lake Annecy (45°53′ N, 6°08′ E) and Lake Bourget (45°43′ N, 5°52′ E) are two geographically close French peri‐alpine lakes (Table 1). They possess similar physical characteristics (e.g., size and maximum and average depths) but differ in trophic dynamics, biodiversity and fisheries management (Changeux et al., 2024; Jacquet et al., 2014). Lake Annecy is oligotrophic, with total phosphorus concentrations being less than 10 μg P L^−1^ since 1994 (Frossard et al., 2024). Lake Bourget experienced a period of strong eutrophication (120 μg P L^−1^ in 1983) followed by a reoligotrophication phase, and since the early 2020s, most indicators classify it as oligotrophic (Jenny et al., 2024). Phosphorus concentrations from recent years are shown in Figure S1.

**TABLE 1:** Characteristics of the study lakes.

Specification	Annecy	Bourget
Area (ha)	~2700	~4450
Watershed (km^2^)	251	588
Volume (km^3^)	~1.13	~3.6
Length (km)	14.6	18
Width (km)	0.80 to 3.35	1.60 to 3.50
Altitude (m)	446	231.5
Average depth (m)	41.5	85
Maximum depth (m)	65	145
Mean winter concentrations of total phosphorus (μg TP. L^−1^)	Under 10 since 1990 (Frossard et al., 2024)	From 120 in 1983 to under 10 since 2011 (Jenny et al., 2024)

Approximately 15 fish species are found in Lake Annecy, 20 in Lake Bourget, and perch is the numerically dominant species in both lakes (Frossard et al., 2024; Jenny et al., 2024). Based on standardized gillnetting surveys (OLA‐IS, AnaEE‐France, INRAE of Thonon‐les‐Bains, SILA, CISALB, Rimet et al., 2020), European perch accounted for 83% of the individuals captured in both the benthic and pelagic gillnets in Lake Annecy and 66% in Lake Bourget (Frossard et al., 2024; Jenny et al., 2024).

### 2.2 Estimation of YOY perch density

Following standard protocols (CEN, 2014) and recommendations (Draštík et al., 2017), hydroacoustic surveys were performed annually at night during late summer (mid‐September for Lake Annecy and end of September to early October for Lake Bourget) from 2011 to 2023 using scientific echo‐sounders (Guillard et al., 2006). Initially, a split‐beam EK60 Simrad (70 kHz; Simrad Kongsberg Maritime AS, Horten, Norway) was used, and most recently, surveys were performed with a split‐beam EK80 Simrad (120 kHz). Hydroacoustic data collected using these two frequencies and generations of sounders yield comparable results (De Robertis et al., 2019; Guillard et al., 2014; Mouget et al., 2019; Rautureau et al., 2022). Detailed protocols are provided in Guillard et al. (2006) for Lake Annecy and in Yule et al. (2013) for Lake Bourget, as well as in the annual survey reports for both lakes (Frossard et al., 2024; Jenny et al., 2024). These surveys provide an indicator of fish density (number of fish. ha^−1^) in the upper water layer above the thermocline, where YOY perch predominantly reside (Guillard et al., 2006; Yule et al., 2013) but also, to a lesser extent, other species such as young roach. It is not possible to identify the species of fish detected by hydroacoustics; however, in scientific pelagic gillnet surveys conducted during the same period, YOY perch consistently accounted for most fish caught above the thermocline, averaging 93% in Lake Annecy and 74% in Lake Bourget (OLA‐IS, AnaEE‐France, INRAE Thonon‐les‐Bains, SILA, CISALB, Rimet et al., 2020, Frossard et al., 2024; Jenny et al., 2024). Although these proportions fluctuated in years with low overall pelagic catches, the median values remained high at 96% in Annecy and 77% in Bourget. Consequently, fish density estimates from these surveys provide a reliable proxy for the annual strength of the YOY perch cohort throughout the studied period (Guillard et al., 2006; Sotton et al., 2011), referred to in the text as YOY perch density.

### 2.3 Determination of developmental stages

To investigate the relationships between environmental variables and YOY perch density, we defined the duration of the critical stages of the perch life cycle and selected the main environmental variables influencing perch density during this period based on the literature (Craig, 2000; Fraz et al., 2024; Gillet & Dubois, 2007; Schmitz & Sepulveda Villet, 2021; Wang & Eckmann, 1994). The timing of the early developmental stages of perch in Lakes Annecy and Bourget was estimated from the spawning period in Lake Geneva. These three lakes are geographically close, with Lake Geneva located approximately 40 km from Lake Annecy and 60 km from Lake Bourget. The timing of early development was also inferred from degree days (°C Day), defined as the cumulative sum of daily mean temperatures above 0°C, which is used to quantify developmental rates in fishes (Neuheimer & Taggart, 2007).

In peri‐alpine lakes, perch spawning is triggered by water temperature in the littoral zone (Gillet & Dubois, 2007). For instance, in Lake Geneva, the date at which 50% of eggs are deposited is correlated with the date at which the surface water temperature reaches 12°C (Gillet & Dubois, 2007). Based on the method described in Gillet and Dubois (2007), the phenology of perch reproduction in Lake Geneva has been studied continuously since 1986 (Goulon et al., 2024).

For each year, records of spawning dates and the associated number of eggs laid are available for Lake Geneva, along with daily modelled surface water temperatures (Sharaf et al., 2023). In this study, we analysed 36 years of perch monitoring records and found that egg laying is concentrated between 10.5°C and 13.3°C, which are the temperatures at which 10% and 90% of the total spawning events, respectively, have occurred (Figure S2). The maximum spawning activity occurred at 11.5°C. Based on this analysis, we estimated the spawning periods of perch in peri‐alpine lakes as the dates when the surface water temperature reached 10.5°C (10% of the number of eggs deposited) and 13.3°C (90% of the number of eggs deposited). These temperature thresholds are valid only for lakes within the same geographical region, as perch spawning temperatures vary from north to south across the geographical distribution of the species (Thorpe, 2011).

Considering incubation periods of 13–14 days at 14°C and 15–16 days at 12°C (Guma'A, 1978), perch eggs would require 187.5 ± 8 degree‐days. The threshold was set at 187.5 degree‐days, and the duration of the larval stage was defined as 33 days after hatching based on literature values (Vlavonou, 1996).

### 2.4 Parameters considered for the embryonic stage

**FIGURE 1:**
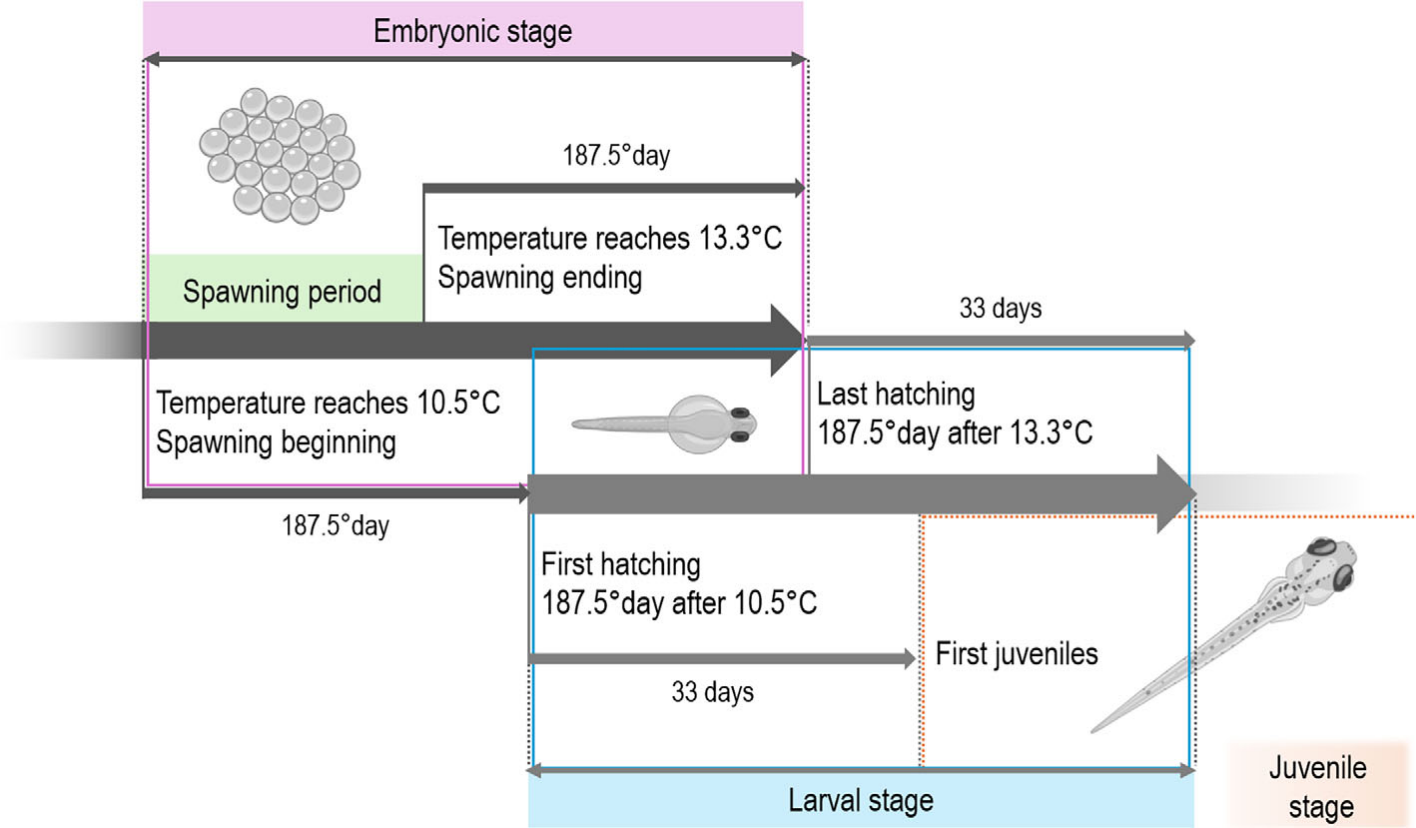
Summary diagram of the early developmental stages of perch based on our survey data and the literature (Guma'A, 1978; Vlavonou, 1996).

The embryonic stage begins when the surface temperature reaches 10.5°C, which corresponds to the start of the spawning period (Figure 1). Even if the first eggs laid become larvae approximately 187.5 degree‐days after the date of spawning, the embryonic phase does not stop at that point. To consider the theoretical incubation phase of all the spawns, we identified the time at which the incubation phase of the last eggs laid ended, that is, 187.5 degree‐days after a temperature of 13.3°C was reached for surface water. The different dates for the beginning and end of the embryonic and larval periods for each lake and each year are summarized in Table S1.

#### 2.4.1 Temperature data and thermal indices

In situ surface temperature data were acquired using Tinytag or Hobo Onset underwater data loggers between 2011 and 2023 for Lakes Annecy and Bourget, respectively. In Lake Annecy, temperatures were measured in the littoral zone every half‐hour at 4 stations between 0.5 and 1 m depth (data from the SILA, Syndicat Mixte du Lac d'Annecy—Lake Annecy Mixed Syndicate). In Lake Bourget, measurements were recorded every minute at a single monitoring station at a depth of 1.2 m with a continuous temperature record covering the entire study period (data from the CISALB, Comité Intercommunautaire pour l'Assainissement du Lac du Bourget—Intercommunal Committee for the Sanitation of Lake Bourget). Although additional stations were available in Lake Bourget, the time series were not long enough and were not included. Nevertheless, surface temperatures were comparable among the stations within each lake, with no significant differences observed. Furthermore, since perch generally reproduce in coastal habitats, most often in the top few meters of the water column (Gillet & Dubois, 2007), the temperature measurements used here are highly representative of spawning conditions.

The mean daily surface water temperature was calculated at each station and subsequently averaged across the stations for Lake Annecy. In Annecy, the number of active monitoring stations has varied over time, with between one and four measurements available per day depending on the operational conditions. For periods with missing in situ measurements (e.g., during probe maintenance, vandalism and data loss), which represented 25% of the April–June sequences in Annecy and 7% in Bourget (Figure S3), daily surface temperatures were reconstructed using simulations from the two‐layer semiempirical Ottosson–Kettle–Prats Lake Model (OKPLM) (Prats & Danis, 2019; Sharaf et al., 2023). The model simulates temperatures based on the geomorphological characteristics of the lake as input and is forced with meteorological data, including air temperature and solar radiation. In this study, simulations were conducted using model parameters calibrated with observation data from the Observatory of LAkes (OLA) (Rimet et al., 2020) and meteorological data from SAFRAN, Système d'Analyse Fournissant des Renseignements Adaptés à la Nivologie – Analysis System Providing Information Tailored to Snow Science (Durand et al., 1993).

Based on these surface temperature data, we calculated four indices to characterize the thermal conditions relevant to embryonic development. The first index, the mean embryonic thermal (MET) index, corresponds to the mean temperature of the embryonic phase and reflects average incubation conditions (Guma'A, 1978; Hokanson & Kleiner, 1974; Linløkken, 2023). The second index, the embryonic thermal amplitude (ETA) index, represents the thermal amplitude during incubation and is calculated as the difference between the highest and lowest daily mean temperatures to quantify thermal variability during development (Schmitz & Sepulveda Villet, 2021; Van De Hey et al., 2013).

The third index, the embryonic thermal index (ETI), was developed in this study to account for differences in daily mean temperature during egg incubation, allowing discrimination between years with stagnant or highly variable temperatures and years with stable warming conditions. This index is based on the known sensitivity of fish embryos to short‐term temperature fluctuations (Clady, 1976; Clady & Hutchinson, 1975; Schmitz & Sepulveda Villet, 2021; Vasseur et al., 2014). The ETI is calculated as follows:

$$\mathrm{ETI}=\sum_{i=1}^{n}\mathrm{TV}$$

where temperature variation (TV) is the difference between two consecutive daily mean temperatures in°C, with one value from the first to the last day of incubation (*n*). Further details on the calculation (Table S2) and the advantages of ETI (Figure S4) are available in Section S5. The reliability of the ETI calculation based on fixed temperature thresholds was also assessed using phenological data on reproduction observed on Lake Geneva (Figure S5), available in Section S6.

Finally, the thermal embryonic variation (TEV) index is derived from the ETI using absolute TV values and is calculated as follows:

$$\mathrm{TEV}=\sum_{i=1}^{n}|\mathrm{TV}|$$

where |TV| is the absolute daily TV (°C) with one value from the first to the last day of incubation (*n*), providing a measure of cumulative thermal instability during embryonic development.

#### 2.4.2 Wind speed index

During the embryonic phase, in addition to temperature, wind is considered as a potential factor influencing incubation success (Bourinet et al., 2023; Clady & Hutchinson, 1975). To capture its influence, the embryonic wind condition (EWC) index was defined as the mean wind speed (m/s) during the embryonic phase. For additional information on the wind speed data, refer to the Supporting Information (Section S7).

### 2.5 Parameters considered for the larval phase

The larval phase partially overlaps with the embryonic phase; as the first larva emerges from the eggs, other embryos are still developing (Figure 1). The larval phase thus begins 187.5 degree‐days after the surface temperature reaches 10.5°C, corresponding to the first hatch, and ends 33 days after the surface temperature reaches the threshold of 13.3°C plus 187.5 degree‐days for hatching, corresponding to the date when the last larva reaches the juvenile stage. The data are summarized in Table S1.

#### 2.5.1 Temperature data and indices

After hatching, larvae remain strongly influenced by temperature, which impacts individual growth (Craig, 2000; Linløkken, 2023). The four thermal indices originally used for the embryonic phase were adapted for the larval phase. The mean larval thermal (MLT) index corresponds to the mean temperature during the larval phase, and the larval thermal amplitude (LTA) index represents the difference between the maximum and minimum temperatures during larval development. Like the ETI, the larval thermal (LTI) index sums the daily TV during the larval phase:

$$\mathrm{LTI}=\sum_{i=1}^{n}\mathrm{TV}$$

Finally, the thermal larval variation index (TLV) is similar to the LTI but uses absolute values of TV to represent the total thermal variations experienced by the larvae during their 33 days of larval development:

$$\mathrm{TLV}=\sum_{i=1}^{n}|\mathrm{TV}|$$

#### 2.5.2 Other environmental parameters

In addition to temperature, other environmental parameters were considered for the larval phase. These include the larval wind condition index (LWC), mean wind speed during the larval period, zooplankton prey availability at first feeding indices (i.e., rotifers, zai_rot; copepods zai_cop; Cladocera, zai_clad; and all available prey zai_all) (Craig, 2000; Masson et al., 2001; Wang, 1994) and adult perch abundance as genitor stock indicator (Gen index) and as intraspecific competition or predation proxies (Pred index) (Craig, 1978, 2000; Gillet & Dubois, 2009). Detailed descriptions of these parameters are provided in the Supporting Information (Sections S8 and S9).

To assess the lake trophic dynamics and nutrient availability, the mean concentrations of total phosphorus (TP) and orthophosphate in the 0–15 m water layer from April to June (2011–2023) were also examined. The TP and orthophosphate concentrations were obtained from OLA survey data (Rimet et al., 2020) and correspond to depth‐weighted means expressed as mg P L^−1^ (elemental phosphorus), determined by the molybdenum‐blue method after sulfuric acid digestion (EPA 365.3). Dissolved inorganic phosphorus concentrations from orthophosphate are reported as μg P L^−1^ throughout this study. These nutrient concentrations are used as proxies for nutrient availability (Schindler, 1978), which may influence the spring development of the primary trophic network, support zooplankton production and ultimately sustain *P. fluviatilis* larvae during their critical early life stages (Wang & Eckmann, 1994). During this period, the TP concentrations ranged from 4 to 9 μg L^−1^ in Lake Annecy and from 7 to 17 μg L^−1^ in Lake Bourget (Figure S1).

### 2.6 Statistical analysis

Statistical analyses were performed in RStudio, Version 2025.05.0, using the R statistical environment (R Core Team, 2025). Generalized additive models (GAMs) were used to model the nonlinear functional relationship between our set of explanatory variables and the annual YOY perch density for each lake using the mgcv (Wood, 2022) and gratia (Simpson & Singmann, 2022) packages, following the methods described in Simpson and Anderson. Given the number of observations available, to avoid overfitting and preserve degrees of freedom, we used simple covariate models. Each GAM was modelled using thin‐plate regression splines as the basis spline, and the degree of smoothness was determined via restricted maximum likelihood (REML). A total of 18 explanatory variables were tested (Table S3). The model's residuals were checked using the *appraise* function from the *gratia* package (Simpson, 2024), PACF diagnostics and standard tests (Shapiro, Breusch–Pagan, Durbin‐Watson). For the environmental factors that were identified as significant in both studied lakes (*p*‐value of the GAM <0.05), hierarchical GAMs (HGAMs) were subsequently used to assess whether the YOY perch responded similarly to these factors in both lakes (Pedersen et al., 2019). The two datasets were combined, resulting in a total of 22 observations, allowing assessment of shared effects on YOY perch abundance in both lakes. To this end, different types of models (G, S, GS, I and GI) were compared to determine the best fit using the method described in Pedersen et al. (2019). The equations used for each model are shown in Table 2. Two categories of models were applied: nonglobal trend models (S and I models) and shared global trend models (G, GS and GI models). For the nonglobal trend models, the ‘S’ model assumes that the lake‐specific smoothers have the same wiggleness, whereas for the ‘I’ model, both trends and wiggleness are lake specific. Note that for the latest model, the tensor product allows each of the smoothers to have a lake‐specific wiggleness because the ‘Lake’ variable appears to have a random effect structure (bs = ‘re’). Similarly, for the models with a shared global trend, the ‘G’ model includes only one global smoother for both lakes, whereas the ‘GS model’ includes a common smoother as well as a lake‐specific smoother that has the same wiggleness. In the formula of the GS model, the explanatory variable (bs = ‘tp’) corresponds to the common smoother as for the G model, and s(explanatory variable, Lake, bs = c (‘tp’,’fs’)) corresponds to the lake‐specific smoothers. Finally, the ‘GI model’ combines the global trend smoother with a group‐level smoother where lakes can have specific smoothers with different wiggleness values. For this most complex model, the formula is s(explanatory variable, bs = ‘tp’), which is a primary parameter for the shared trend; another s(explanatory variable, by = Lake, bs = c (‘tp’, ‘fs’)) corresponds to a second lake‐scale smoother and a final s(Lake, bs = ‘re’), which allows the wiggleness to be adapted to each lake by introducing the Lake variable as having a random effect structure.

### 2.7 Ethical statement

The use of experimental animals was deemed superfluous in the present study. The data collected were obtained from measurements taken in the natural environment on natural fish populations not affected by the study. The use of hydroacoustics, a non‐intrusive method, made this possible (more details are provided in Section 2.2).

## 3 RESULTS

### 3.1 Annual YOY perch density

YOY perch densities varied strongly from year to year and between lakes. The minimum was recorded at 128 individuals per hectare for Annecy in 2023 and 149 individuals per hectare for Bourget in 2022 (Figure S7). The maximum was 5585 individuals per hectare for Annecy in 2019 and 6833 for Bourget in 2020. On average, there was a tenfold difference between minimum and maximum densities.

### 3.2 Indices by lake

Simple GAMs using a single covariate were processed with the formula: Density (of YOY perch) ~ s(Explicative variable, bs = ‘tp’). The results of the GAMs are shown in Table S3. Among the 18 factors per lake model combinations tested, only 6 were found to have a significant effect on YOY perch density, and 5 were related to temperature.

**FIGURE 2:**
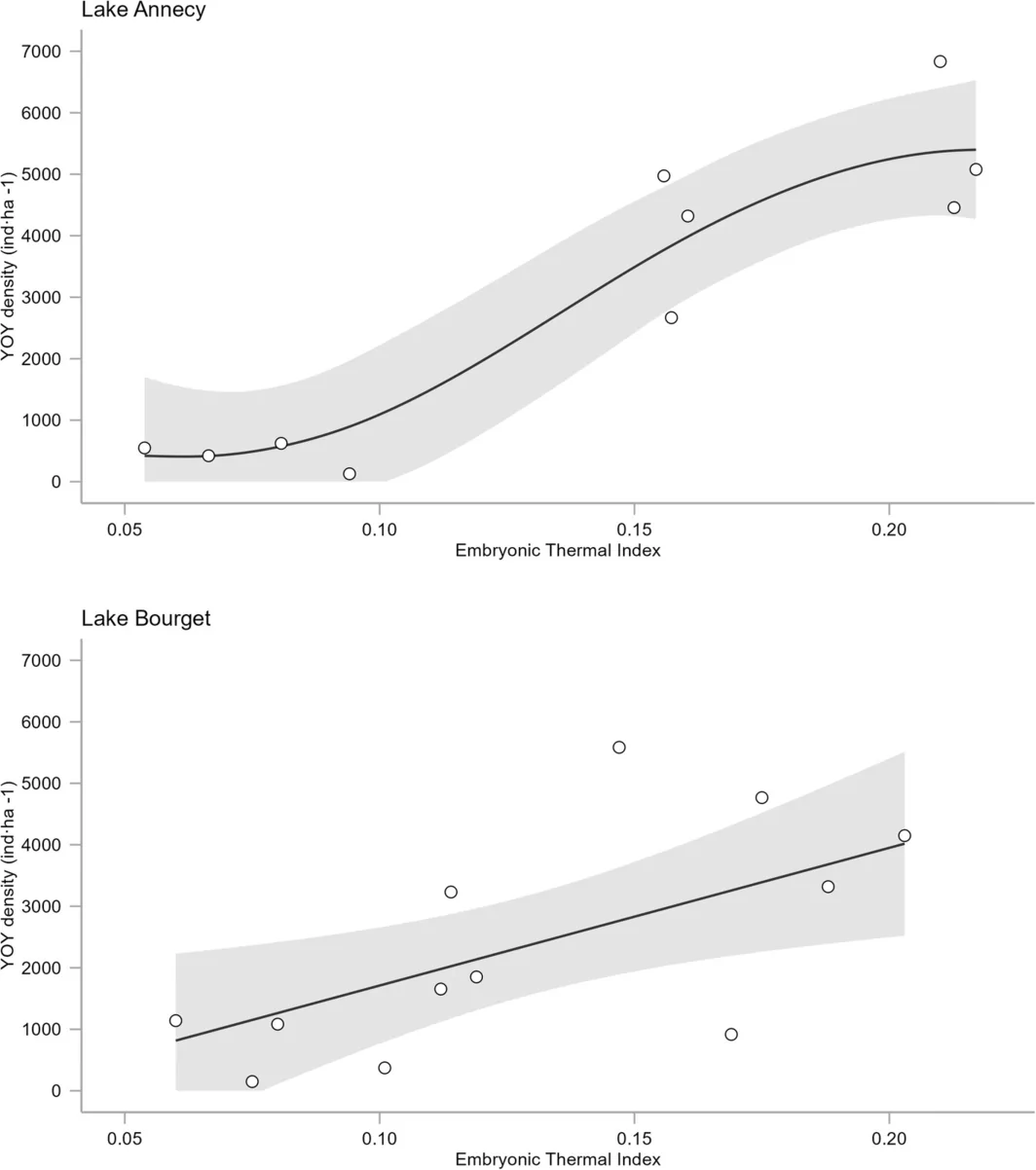
Effects of the embryonic thermal index (ETI) on young‐of‐the‐year (YOY) density in Lake Annecy (top) and Lake Bourget (bottom), single covariate generalized additive models (GAMs).

In Lake Annecy, four factors influenced the YOY density (Table S3), and three were significant and related to temperature during the embryonic phase, namely, the MET, ETA and ETI. With respect to these three factors, the GAMs explained 80.5%, 88.8% and 87.6% of the deviance, respectively. All three exhibited a similar trend. The higher the explanatory variable is, the higher the density of YOY perch in late summer (Figure 2). However, for ETA, the conditions for homoscedasticity of the model's residuals were not satisfied; as such, the model of YOY density using the ETA factor could not be validated. During the larval development stage, another significant thermal index, LTA, was found to significantly influence YOY density variability during the larval phase. LTA explained 51.6% of the variability in density. The higher the LTA, the lower the density.

Among the three models found to be relevant for Lake Bourget, one involved a temperature‐based predictor during the embryonic stage (Table S3). Indeed, the ETI explained 43.9% of the variation in density for Lake Bourget. The variance explained was lower than that explained in Lake Annecy, but the relationships were similar. The higher the ETI, the more YOY perch are present at the end of summer (Figure 2). Another factor not related to the temperature index, the Gen index of the genitor stock, was also significant in Bourget. Explaining 32.7% of the variation in density, the relationship seemed to indicate that when more genitors were present, fewer YOY perch were found at the end of the summer (Figure S6). Conversely, the intraspecific predation index, Pred, was not significant (*p* = 0.0636) but shows the opposite relationship compared with the Gen index. Indeed, the higher the potential cannibalism, the higher the density of YOY perch.

### 3.3 Comparison of ETI tendencies of the two lakes

**FIGURE 3:**
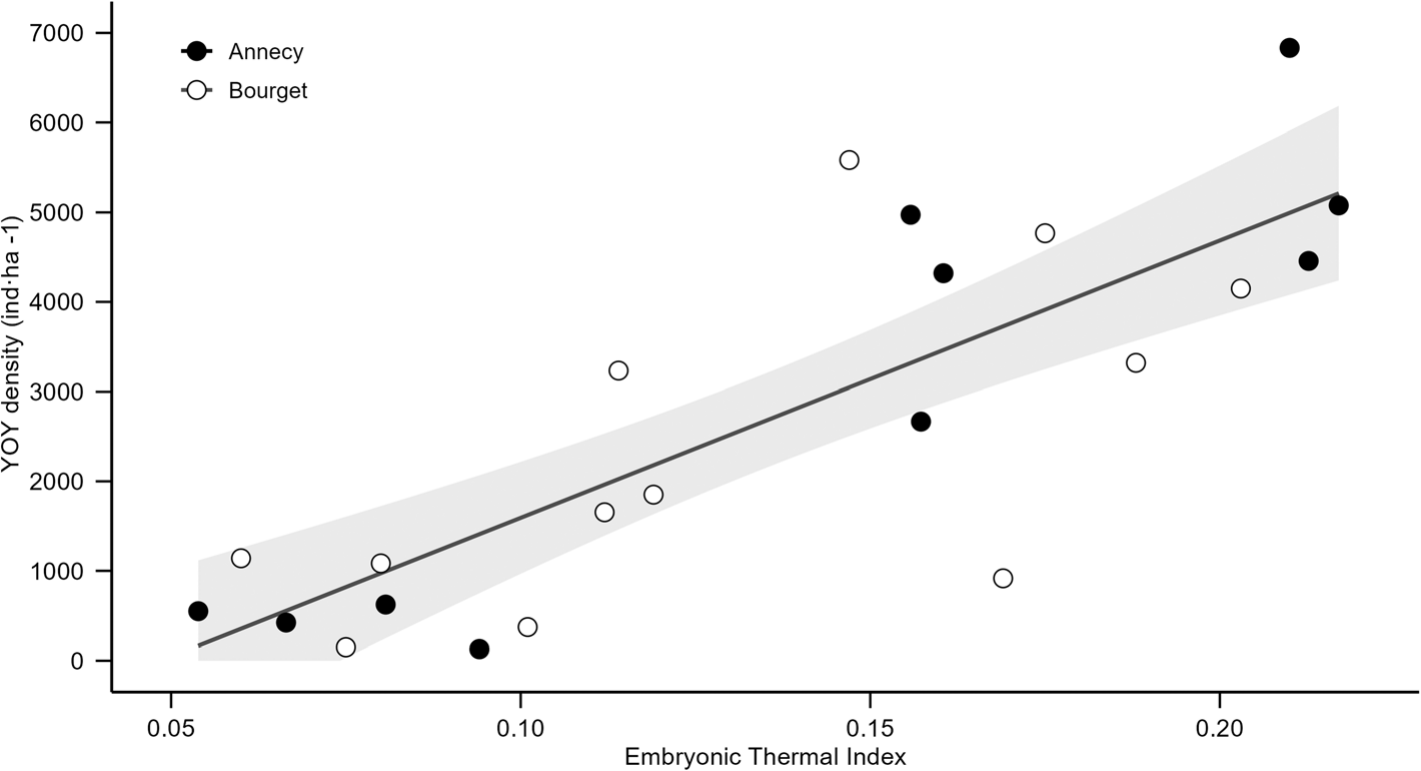
Effect of the embryonic thermal index (ETI) on young‐of‐the‐year (YOY) density in Lakes Annecy (black circles) and Bourget (white circles), according to the hierarchical generalized additive model (GAM) (Pedersen et al., 2019).

The only index that was significant and similar in both lakes was the ETI. Therefore, the next step was to assess whether the relationship between incubation temperature and YOY perch density at the end of the summer was identical in both lakes or lake‐dependent. The close performances of these models, particularly G (Figure 3), GS and GI, which all incorporate a global trend component, suggest that a general positive relationship between embryonic thermal index (ETI) and YOY perch density is shared between the two systems. This interpretation is supported by the overall pattern observed across the models: YOY density consistently increased with increasing ETI values in both lakes. The model GI, which includes lake‐specific smoothers, and model I, which fits entirely separate responses for each lake, were slightly less parsimonious, indicating that although local particularities may exist, the dominant effect of embryonic temperature does not significantly differ between lakes.

**TABLE 2:** Hierarchical generalized additive model (HGAM) results with the combined data from the two lakes. For each model (Pedersen et al., 2019), the global trend, akaike information criterion (AIC), degree of freedom (df), deviance explained, formula and *p* value by smooth terms (signif. codes: ***p < 0.001; **p < 0.01; *p < 0.05; *p* < 0.1) are detailed.

Type	Global trend	AIC	df	Deviance explained	Formula	Smooth terms	*p* value
*S*	No	379.53	2.99	66.8%	Density ~ s(ETI, bs = ‘tp’)	s(ETI)	2.9e‐06 ***
*G*	Yes	379.53	3.00	66.8%	Density ~s(ETI, bs = ‘tp’) + s(Lake, bs = ‘fs’)	s(ETI)	2.9e‐06 ***
						s(Lake)	0.606
*GI*	Yes	379.86	3.42	67.5%	Density ~s(ETI, bs = ‘tp’) + s(ETI, by = Lake, bs = c(‘tp’,’fs’)) + s(Lake, bs = c‘re’)	s(ETI)	2.7e‐05 ***
						s(ETI):Lake Annecy	0.525
						s(ETI):Lake Bourget	0.805
						s(Lake)	0.602
*GS*	Yes	379.86	3.42	67.5%	Density ~ s(ETI, bs = ‘tp’) + s(ETI, by = Lake, bs = ‘fs’)	s(ETI)	9.6e‐06 ***
						s(ETI):Lake Annecy	0.254
						s(ETI):Lake Bourget	0.599
*I*	No	381.35	5.22	70.5%	Density ~ s(ETI, by = Lake, bs = c(‘tp’, ‘fs’)) + s(Lake, bs = ‘re’)	s(ETI):Lake Annecy	6.9e‐05 ***
						s(ETI):Lake Bourget	0.0037 **
						s(Lake)	0.618

## 4 DISCUSSION

### 4.1 Dominant role of ETI values

Despite covering only a decade, our results clearly demonstrate the importance of temperature‐based predictors, particularly the ETI, in explaining year‐to‐year variations in the density of YOY perch. These results support our hypothesis that fine‐scale thermal indices based on phenology, such as the ETI, better explain recruitment variability than seasonal temperature measurements. Consistent with this finding, a strong correlation was observed between the ETI and the density of YOY perch at the end of summer across years in both lakes. These findings suggest that recruitment success is largely determined by abiotic conditions. Indeed, ETI was the only factor consistently associated with recruitment patterns across both systems. High ETI values, reflecting steady warming with limited day‐to‐day variability, were associated with higher YOY densities, whereas low ETI values, driven by stagnant or fluctuating thermal conditions, coincided with reduced recruitment. ETI and TEV capture different aspects of thermal variability. TEV measures the magnitude of temperature fluctuations; however, it ignores their direction. In contrast, ETI considers whether temperatures are increasing or decreasing, thus reflecting the thermal trajectory during incubation. This distinction likely explains why, unlike TEV, ETI explained recruitment variability, as embryonic development depends more on consistent warming than on variability alone.

In Lake Bourget, ETI explained 44% of YOY perch density variance, and in Lake Annecy, it explained up to 88%, emphasizing the dominant role of ETI values in shaping recruitment dynamics. These findings are consistent with those of earlier studies on yellow perch showing that egg mortality increases under stagnant or decreasing temperatures during embryonic development (Clady, 1976; Guma'A, 1978). Incubation near optimal temperatures has also been linked to larger larval size at hatching and improved early survival (Wang, 1994). More broadly, spring temperatures have been shown to positively influence both larval and juvenile densities (Dembkowski et al., 2017; Pope et al., 1996; Ward et al., 2004), in line with our results for Eurasian perch. Hierarchical modelling further confirmed that this positive thermal effect is shared across lakes and outweighs system‐specific differences. The most parsimonious models consistently supported a common thermal recruitment relationship, indicating that warming during the incubation phase benefits perch recruitment regardless of local environmental conditions. Moreover, the difference in explained variance by ETI between Lake Bourget (44%) and Lake Annecy (88%) could be due to the lower percentage of YOY perch in the upper layer of Lake Bourget (74%) compared to Lake Annecy (93%). As a result, the proxy for YOY perch density would be less reliable in Lake Bourget, resulting in a lower response to the ETI.

During the spawning period, thermal stratification is still weak, allowing wind‐driven mixing or localized upwelling in littoral areas (Gillet & Dubois, 1995), which can bring colder water to the surface and increase embryonic mortality (Clady, 1976; Newsome & Aalto, 1987). This likely explains the abrupt temperature drops observed in some years. Wind‐driven currents may also displace egg ribbons into suboptimal habitats (Čech et al., 2011; Clady & Hutchinson, 1975), further increasing mortality. Although we did not distinguish between mechanical and thermal effects, it is likely that both contribute to variability in interannual recruitment. Conversely, rapid spring warming can increase recruitment by accelerating egg development and reducing exposure time to predation, dispersal and physical disturbance (Clady, 1976; Clady & Hutchinson, 1975; Newsome & Aalto, 1987). It may also limit thermal stress, reduce developmental abnormalities (Hokanson & Kleiner, 1974), and improve match–mismatch dynamics with zooplankton peaks (Busch et al., 1975; Pitlo, 2002).

### 4.2 Timing of spring warming

Some studies have reported that perch recruitment is strongest when warm conditions persist throughout both the embryonic and larval stages (Clady, 1976; Kipling, 1976). According to the growth hypothesis (Anderson, 1988), extended warm conditions from spring to summer can influence year–class strength, as observed in *P. fluviatilis* and *P. flavescens* populations (Craig & Kipling, 1983; Dembkowski et al., 2017). Geographically, Lakes Annecy and Bourget are in the southern part of the species' natural distribution, and summer surface temperatures rarely fall below 14°C, consistently exceeding the lower growth threshold of 13.5°C (Power & Van Den Heuvel, 1999). Spring–summer temperatures were generally above levels limiting larval and juvenile development. These observations indicate that post‐hatching thermal conditions were unlikely to constrain recruitment.

Taken together, these results show that recruitment depends not only on temperature magnitude but also on the timing and stability of warming. In Lake Bourget, a recent shift towards earlier and more rapid spring warming was observed, with spawning occurring 14–20 days earlier than average in March 2020 and 2022. In both years, temperatures rapidly reached optimal values (~11°C) before abruptly declining, leading to low ETI values and reduced YOY densities. These results suggest that early but unstable warming events can negatively affect recruitment, likely by exposing embryos to renewed cold stress (Van De Hey et al., 2013) or disrupting developmental synchrony (Cushing, 1990; Durant et al., 2007). Therefore, beyond overall warming, the temporal dynamics of spring temperature are critical for recruitment success, and increasing climate variability may fundamentally alter recruitment patterns.

### 4.3 Thermal conditions during the larval stage

During the larval phase, a significant effect was detected only in Lake Annecy. LTA, which represents the temperature range experienced during this period, was the only index significantly related to YOY density, with higher LTA associated with lower recruitment. A similar negative pattern was also observed for Lake Annecy for the LTI, although this relationship was marginally nonsignificant.

LTA was also significantly correlated with ETI, indicating that years with low ETI values tend to be followed by higher thermal variability during the larval stage. In practice, low ETI values were consistently associated with both reduced YOY density and elevated LTA, as surface temperatures eventually reached similar levels (~20°C) by June in all years. Notably, high LTA values often coincided with years that included modelled temperature data, which may slightly inflate thermal variability due to smoothing effects. Conversely, low LTA values corresponded to years with warm and stable conditions from the start of the larval phase, which were more favourable for recruitment.

### 4.4 Role of trophic factors and food availability

Temperature also influences perch energetics and feeding dynamics by regulating both food intake and prey phenology, potentially leading to match–mismatch dynamics between larvae and zooplankton (Craig, 1978, 1987). However, the critical window for prey availability is short (Wang & Eckmann, 1994), and our dataset lacked high‐frequency zooplankton data, preventing a precise evaluation of this mechanism. Indeed, zooplankton abundance was not a significant predictor of YOY density in either lake in our analysis. However, laboratory work has demonstrated that prey size, type and abundance strongly affect larval and juvenile perch growth and survival (Arts & Sprules, 1989; Graeb et al., 2004; Romare, 2000). Slow growth during early summer can extend the period of vulnerability to predation, decreasing survival (Bailey & Houde, 1989; Miller et al., 1988). For instance, in Lake Annecy, zooplankton do not appear to limit growth, possibly because of sufficient resource availability in this oligotrophic system (Perga et al., 2009; Perrier et al., 2012). In contrast, other systems have shown strong links between prey availability and perch dynamics (Dubois et al., 2008). In our study, the zooplankton indices showed no temporal trend but were correlated with nutrient concentrations, suggesting that they may act as proxies of trophic status rather than direct drivers of recruitment (Alric et al., 2013).

### 4.5 Influence of adult stock and predation

Adult stock effects were difficult to interpret. In Lake Bourget, the Gen index (spawning stock for year *n* + 1) was negatively associated with YOY density, whereas the Pred index (potential cannibalism) showed a nonsignificant positive trend; these patterns likely reflect methodological limitations rather than true biology. Estimates of adult abundance were derived from gillnet surveys, which are prone to selectivity bias and undercatch (Olin et al., 2009). The link between fishing mortality and commercial fishing was also not considered. Neither index was significant in Lake Annecy.

Predation affects perch recruitment, including cannibalism (Craig, 1978, 2000; Dembkowski et al., 2017; Gillet & Dubois, 2009). However, in our systems, the effects of adult stock and cannibalism appear to be secondary, partly due to data limitations. Other predators (e.g., pike) were excluded due to a lack of detailed data; although predation effects can vary across systems, they can sometimes indirectly promote recruitment by reducing competition (Dembkowski et al., 2017). Future work should aim to improve adult stock indicators to clarify the roles of cannibalism and spawning stock on YOY dynamics.

### 4.6 Methodological limitations and contributions to the field

We were unable to detect any correlations between, on the one hand, the availability of zooplankton and parameters related to adult stocks and, on the other hand, the density of YOY perch. Furthermore, differences in data quality and resolution are reflected in contrast with temperature‐based indices. Whereas thermal variables were derived from continuous, high‐frequency measurements, biological variables were based on more limited and indirect estimates. The zooplankton data were collected at a much lower temporal resolution (monthly), potentially causing critical short‐term dynamics to be overlooked. Adult perch abundance was inferred from gillnet surveys, which are subject to selectivity bias and undercatch. These limitations likely reduced the explanatory power of biological variables regarding recruitment.

This study also demonstrates the value of long‐term hydroacoustic monitoring. Such surveys are rare in freshwater systems, especially across multiple lakes over decades (Pollom & Rose, 2016). Our work shows that these techniques provide robust ecological insights and a unique perspective on the environmental drivers of fish recruitment in lakes.

### 4.7 Conclusions and perspectives

This study revealed that the success of perch recruitment primarily depends on the temperature conditions experienced during the embryonic stage. Steady warming with minimal day‐to‐day temperature fluctuations during this critical period results in higher densities of YOY perch. By showing that fine‐scale temperature indicators based on phenology outperform conventional seasonal averages, our results emphasize the importance of aligning environmental descriptors with biological calendars. In the context of current global climate change, this mechanistic understanding provides a valuable basis for anticipating how changes in spring temperature regimes might affect recruitment dynamics in lake ecosystems.

## AUTHOR CONTRIBUTIONS

V.C. led the study, performed the analyses and drafted the manuscript. C.Gou. co‐supervised the project, supported the study throughout and contributed to manuscript preparation and writing. J.G. co‐supervised the project, secured funding and contributed to hydroacoustic analyses and writing. C.B. contributed to statistical analyses and manuscript writing. O.A. supported later stages of writing and provided critical revisions. N.S. conducted lake temperature simulations. C.Gil. contributed legacy expertise and background knowledge on Eurasian perch in Lake Geneva. All authors reviewed, edited and approved the final version of the manuscript.

## Supporting information


**Figure S1.** Trends for Lake Annecy (diamond) and Lake Bourget (circle) in total phosphorus (full) and orthophosphorus (empty) from April to June between 0 and 15 m depth for the period 2011–2023 (OLA survey data http://www6.inra.fr/soere-ola © OLA‐IS, AnaEE‐France, INRAE, Thonon‐les‐Bains, SILA, CISALB). To assess the potential influence of lake trophic status and its long‐term evolution on perch recruitment, we selected the mean concentrations of total phosphorus and orthophosphorus between April and June as a proxy. Phosphorus is widely recognized as the most limiting nutrient for primary producers in lake ecosystems and thus plays a key role in structuring trophic dynamics. As such, it can indirectly influence zooplankton abundance at the time of perch larval emergence. During this critical period of first feeding, prey availability is essential for survival. This phosphorus‐based index is therefore of dual interest: it reflects both the trophic status of the two lakes and provides an indirect measure of potential prey availability for larval perch.
**Table S1.** Theoretical date of development phases (E, embryonic phase and L, larval phase). The last two columns, duration, are in days; the second table represents the average by lake.
**Figure S2.** Perch phenology in Lake Geneva. Distribution of the 17.181 spawns collected on artificial spawning ground from 1986 to 2022 across the temperature range (Gillet & Dubois, 2007; Goulon et al., 2024).
**Figure S3.** Type of temperature data used, within grey, the real data measured on site and in black, the modelled data, for Lake Annecy (A) and Lake Bourget (B).
**Table S2.** Incubation period temperatures, Annecy 2012.
**Figure S4.** Temperature profile during incubation and TV fluctuations in Annecy 2012.
**Figure S5.** Comparison of ETI values derived using the two calculation methods: one based on observed phenology and the other on a temperature‐based prediction method using thresholds of 10.5°C and 13.3°C. To validate index calculation methods and the theoretical reproduction/development period, we compared the ETI values applied to Lake Geneva. On this lake, we have empirical spawning data and modelled surface temperatures (Sharaf et al., 2023). It was therefore possible to calculate two different sets of indices. The first set of ETIs was calculated by taking measured values as the start and end of spawning, recorded during monitoring from 2011 to 2022, to correspond to our study period on lakes Annecy and Le Bourget. The second set of ETIs was calculated as explained in this article, that is, using the 10.5°C and 13.3°C thresholds as the start and end of spawning. No significant difference was found between the two approaches (*p*‐value = 0.577). The linear model relating observed and predicted ETI values was highly significant (*p*‐value = 6.81e‐05 ***), with an adjusted *R* ^2^ of 0.82. A two‐factor ANOVA (calculationmethod × year) was also performed. Only the factor year shows significant differences between ETI values (*p*‐value = 0.006**). No significant differences were observed between the ETI values calculated on real phenology data and on spawning periods determined based on the surface temperature of Lake Geneva. The ETI, being the most complex index, was the only one validated against observed spawning data. Its consistency supports the assumption that simpler indices are also reliable.
**Figure S6.** Effect of the stock of Genitor index (Gen) on YOY density in Lake Bourget, single covariate GAMs.
**Figure S7.** Annual YOY perch density (number of fish detected per hectare, estimated by hydroacoustics) in Lakes Annecy and Bourget.
**Table S3.** Single covariate GAMs results for the 18 variables: mean thermal index (MET), embryonic thermal amplitude (ETA), embryonic thermal index (ETI), thermal embryonic variation (TEV), embryonic wind condition (EWC), mean larval thermal index (MLT), larval thermal amplitude (LTA), larval thermal index (LTI), thermal larval variation (TLV), larval wind condition (LWC), zooplankton abundance indices (rotifers, zai_rot; copepods, zai_cop; cladocerans, zai_clad; and all prey, zai_all), genitor stock index (Gen), predation index (Pred), total phosphorus index (phos_tot) and orthophosphate index (orthophos). For each model, the lake dataset used was specified, as were the number of statistical individuals (n), the explained variance, the *p*‐value of the model (signif. codes: *** *p* < 0.001; ** *p* < 0.01; * *p* < 0.05; *p* < 0.1) and the development phase related to the index.
**Section S5.** Example of ETI calculation (Lake Annecy, 2012).
**Section S6.** Validation of ETI calculation method.
**Section S7.** Wind parameter during the embryonic and larval phases.
**Section S8.** Zooplankton abundance during the larval phase.
**Section S9.** Spawner stock and cannibalism.

## Data Availability

The data that support the findings of this study are available from the corresponding author upon reasonable request.

## References

[jfb70541-bib-0001] Alric, B. , Jenny, J.‐P. , Berthon, V. , Arnaud, F. , Pignol, C. , Reyss, J.‐L. , Sabatier, P. , & Perga, M.‐E. (2013). Local forcings affect lake zooplankton vulnerability and response to climate warming. Ecology, 94(12), 2767–2780. 10.1890/12-1903.1 24597223

[jfb70541-bib-0002] Anderson, J. T. (1988). A review of size dependent survival during pre‐recruit stages of fishes in relation to recruitment. Journal of Northwest Atlantic Fishery Science, 8, 55–66. 10.2960/J.v8.a6

[jfb70541-bib-0003] Arts, M. T. , & Sprules, W. G. (1989). Use of enclosures to detect the contribution of particular zooplankton to growth of young‐of‐the‐year yellow perch (*Perca flavescens* Mitchell). Oecologia, 81(1), 1. 10.1007/BF00377004 28312151

[jfb70541-bib-0004] Ayala, M. D. , Lago, M. J. , & Cal, R. (2015). Body and muscle growth of pre‐larvae and larvae of turbot, *Scophthalmus maximus* L., reared at three different temperatures. Open Journal of Animal Sciences, 5(4), 402–410. 10.4236/ojas.2015.54042

[jfb70541-bib-0005] Bailey, K. M. , & Houde, E. D. (1989). Predation on eggs and larvae of marine fishes and the recruitment problem. In J. H. S. Blaxter & A. J. Southward (Eds.), Advances in marine biology (Vol. 25, pp. 1–83). Academic Press. 10.1016/S0065-2881(08)60187-X

[jfb70541-bib-0006] Bourinet, F. , Anneville, O. , Drouineau, H. , Goulon, C. , Guillard, J. , & Richard, A. (2023). Synchrony in whitefish stock dynamics: Disentangling the effects of local drivers and climate. Journal of Limnology, 82(1), 54–70. 10.4081/jlimnol.2023.2134

[jfb70541-bib-0007] Brucet, S. , Pédron, S. , Mehner, T. , Lauridsen, T. L. , Argillier, C. , Winfield, I. J. , Volta, P. , Emmrich, M. , Hesthagen, T. , Holmgren, K. , Benejam, L. , Kelly, F. , Krause, T. , Palm, A. , Rask, M. , & Jeppesen, E. (2013). Fish diversity in European lakes: Geographical factors dominate over anthropogenic pressures. Freshwater Biology, 58(9), 9. 10.1111/fwb.12167

[jfb70541-bib-0008] Busch, W.‐D. N. , Scholl, R. L. , & Hartman, W. L. (1975). Environmental factors affecting the strength of walleye (*Stizostedion vitreum vitreum*) year‐classes in western Lake Erie. Journal of the Fisheries Research Board of Canada, 32(10), 1960–1970. 10.1139/f75-207

[jfb70541-bib-0009] Čech, M. , Peterka, J. , Říha, M. , Muška, M. , Hejzlar, J. , & Kubečka, J. (2011). Location and timing of the deposition of egg strands by perch (*Perca fluviatilis* L.): The roles of lake hydrology, spawning substrate and female size. Knowledge and Management of Aquatic Ecosystems, 403, 8. 10.1051/kmae/2011070

[jfb70541-bib-0010] Changeux, T. , Boisneau, P. , Stolzenberg, N. , & Goulon, C. (2024). A long‐term overview of freshwater fisheries in France. Reviews in Fish Biology and Fisheries, 34(1), 19–41. 10.1007/s11160-023-09803-5

[jfb70541-bib-0011] Clady, M. D. (1976). Influence of temperature and wind on the survival of early stages of yellow perch, *Perca flavescens* . Journal of the Fisheries Research Board of Canada, 33(9), 1887–1893. 10.1139/f76-241

[jfb70541-bib-0012] Clady, M. D. , & Hutchinson, B. (1975). Effect of high winds on eggs of yellow perch, *Perca flavescens*, in Oneida Lake, New York. Transactions of the American Fisheries Society, 104(3), 3. 10.1577/1548-8659(1975)104<524:EOHWOE>2.0.CO;2

[jfb70541-bib-0013] Craig, J. F. (1978). A study of the food and feeding of perch, *Perca fluviatilis* L., in Windermere. Freshwater Biology, 8(1), 1. 10.1111/j.1365-2427.1978.tb01426.x

[jfb70541-bib-0014] Craig, J. F. (1987). The biology of perch and related fish. Croom Helm.

[jfb70541-bib-0015] Craig, J. F. (2000). Percid fishes: Systematics, ecology and exploitation. Blackwell Science.

[jfb70541-bib-0016] Craig, J. F. , & Kipling, C. (1983). Reproduction effort versus the environment; case histories of Windermere perch, *Perca fluviatilis* L., and pike, *Esox lucius* L. Journal of Fish Biology, 22(6), 6. 10.1111/j.1095-8649.1983.tb04231.x

[jfb70541-bib-0017] Cushing, D. H. (1990). Plankton production and year‐class strength in fish populations: An update of the match/mismatch hypothesis. In J. H. S. Blaxter & A. J. Southward (Eds.), Advances in marine biology (Vol. 26, pp. 249–293). Academic Press. 10.1016/S0065-2881(08)60202-3

[jfb70541-bib-0018] De Robertis, A. , Lawrence‐Slavas, N. , Jenkins, R. , Wangen, I. , Mordy, C. W. , Meinig, C. , Levine, M. , Peacock, D. , & Tabisola, H. (2019). Long‐term measurements of fish backscatter from Saildrone unmanned surface vehicles and comparison with observations from a noise‐reduced research vessel. ICES Journal of Marine Science, 76(7), 7. 10.1093/icesjms/fsz124

[jfb70541-bib-0019] Dembkowski, D. J. , Weber, M. J. , & Wuellner, M. R. (2017). Factors influencing recruitment and growth of age‐0 yellow perch in eastern South Dakota glacial lakes. Fisheries Management and Ecology, 24(5), 5. 10.1111/fme.12240

[jfb70541-bib-0020] Draštík, V. , Godlewska, M. , Balk, H. , Clabburn, P. , Kubečka, J. , Morrissey, E. , Hateley, J. , Winfield, I. J. , Mrkvička, T. , & Guillard, J. (2017). Fish hydroacoustic survey standardization: A step forward based on comparisons of methods and systems from vertical surveys of a large deep lake. Limnology and Oceanography: Methods, 15(10), 10. 10.1002/lom3.10202

[jfb70541-bib-0021] Dubois, J.‐P. , Gillet, C. , Hilgert, N. , & Balvay, G. (2008). The impact of trophic changes over 45 years on the Eurasian perch, *Perca fluviatilis*, population of Lake Geneva. Aquatic Living Resources, 21(4), 401–410. 10.1051/alr:2008051

[jfb70541-bib-0022] Durand, Y. , Brun, E. , Merindol, L. , Guyomarc'h, G. , Lesaffre, B. , & Martin, E. (1993). A meteorological estimation of relevant parameters for snow models. Annals of Glaciology, 18, 65–71. 10.3189/S0260305500011277

[jfb70541-bib-0023] Durant, J. , Hjermann, D. , Ottersen, G. , & Stenseth, N. (2007). Climate and the match or mismatch between predator requirements and resource availability. Climate Research, 33, 271–283.

[jfb70541-bib-0024] Fraz, S. , Laframboise, L. , Manzon, R. , Somers, C. M. , & Wilson, J. Y. (2024). Embryonic and larval development of yellow perch (*Perca flavescens*) and its sensitivity to incubation temperature. Journal of Fish Biology, 105(3), 735–751. 10.1111/jfb.15813 38853288

[jfb70541-bib-0025] Fraz, S. , Thompson, W. A. , Gallucci, M. S. , Afridi, M. , Easwaramoorthy, M. , Hartenstein, P. , Laframboise, L. , Dworkin, I. , Manzon, R. , Somers, C. M. , & Wilson, J. Y. (2025). The effect of early life thermal environment on morphology and growth of yellow perch (*Perca flavescens*). Journal of Fish Biology, 107(3), 712–727. 10.1111/jfb.70066 PMC12463766 40344247

[jfb70541-bib-0026] Frossard, V. , Goulon, C. , Guillard, J. , Hamelet, V. , Jacquet, S. , Lainé, L. , Rautureau, C. , Rimet, F. , & Tran Khac, V. (2024). Suivi de la qualité écologique du lac d'Annecy pour l'année 2023. Carrtel, 1–72. https://hal.science/hal-04682229

[jfb70541-bib-0028] Gillet, C. , & Dubois, J. P. (1995). A survey of the spawning of perch (*Perca fluviatilis*), pike (*Esox lucius*), and roach (*Rutilus rutilus*), using artificial spawning substrates in lakes. Hydrobiologia, 300(1), 409–415.

[jfb70541-bib-0029] Gillet, C. , & Dubois, J. P. (2007). Effect of water temperature and size of females on the timing of spawning of perch *Perca fluviatilis* L. in Lake Geneva from 1984 to 2003. Journal of Fish Biology, 70(4), 4.

[jfb70541-bib-0027] Gillet, C. , & Dubois, J. P. (2009). *Etude de la croissance et de la dynamique des populations de perche (*Perca fluviatilis*) suite aux changements trophiques du lac Léman* .

[jfb70541-bib-0030] Goulon, C. , Vautier, M. , Rogissart, H. , Domaizon, I. , Rautureau, C. , & Guillard, J. (2024). Whitefish (Coregonus sp.) and perch (Perca fluviatilis) spawning in Lake Geneva. CIPEL. https://www.cipel.org/wp‐content/uploads/2024/03/rapport‐scientifique‐2022‐2023‐07‐frai‐coregone‐perche.pdf

[jfb70541-bib-0031] Graeb, B. D. S. , Dettmers, J. M. , Wahl, D. H. , & Cáceres, C. E. (2004). Fish size and prey availability affect growth, survival, prey selection, and foraging behavior of larval yellow perch. Transactions of the American Fisheries Society, 133(3), 3. 10.1577/T03-050.1

[jfb70541-bib-0032] Guillard, J. , Lebourges‐Daussy, A. , Balk, H. , Colon, M. , Jóźwik, A. , & Godlewska, M. (2014). Comparing hydroacoustic fish stock estimates in the pelagic zone of temperate deep lakes using three sound frequencies (70, 120, 200 kHz). Inland Waters, 4(4), 4. 10.5268/IW-4.4.733

[jfb70541-bib-0033] Guillard, J. , Perga, M. E. , Colon, M. , & Angeli, N. (2006). Hydroacoustic assessment of young‐of‐year perch, *Perca fluviatilis*, population dynamics in an oligotrophic lake (Lake Annecy, France). Fisheries Management and Ecology, 13(5), 5.

[jfb70541-bib-0034] Guma'A, S. A. (1978). The effects of temperature on the development and mortality of eggs of perch, *Perca fluviatilis* . Freshwater Biology, 8(3), 3. 10.1111/j.1365-2427.1978.tb01443.x

[jfb70541-bib-0035] Hjort, J. (1914). Fluctuations in the great fisheries of northern Europe, viewed in the light of biological research. Andr. Fred. Høst & Fils.

[jfb70541-bib-0036] Hokanson, K. E. F. , & Kleiner, C. F. (1974). Effects of constant and rising temperatures on survival and developmental rates of embryonic and larval yellow perch, *Perca flavescens* (Mitchill). In J. H. S. Blaxter (Ed.), The early life history of fish (pp. 437–448). Springer.

[jfb70541-bib-0037] Honsey, A. E. , Bunnell, D. B. , Troy, C. D. , Fielder, D. G. , Thomas, M. V. , Knight, C. T. , Chong, S. C. , & Höök, T. O. (2016). Recruitment synchrony of yellow perch (*Perca flavescens*, Percidae) in the Great Lakes region, 1966–2008. Fisheries Research, 181, 214–221. 10.1016/j.fishres.2016.04.021

[jfb70541-bib-0038] Houde, E. (2008). Emerging from Hjort's shadow. Journal of Northwest Atlantic Fishery Science, 41, 53–70. 10.2960/J.v41.m634

[jfb70541-bib-0039] Jacquet, S. , Domaizon, I. , & Anneville, O. (2014). The need for ecological monitoring of freshwaters in a changing world: A case study of lakes Annecy, Bourget, and Geneva. Environmental Monitoring and Assessment, 186(6), 3455–3476. 10.1007/s10661-014-3630-z 24452859

[jfb70541-bib-0040] Jenny, J.‐P. , Avrillier, J.‐N. , Cachera, S. , Costel, C. , Crépin, L. , Iter‐Desgué, O. , Goulon, C. , Guillard, J. , Hamelet, V. , Hustache, J.‐C. , Jacquet, S. , Lainé, L. , Perney, P. , Quétin, P. , Raphy, J. , Rautureau, C. , Rimet, F. , Rasconi, S. , & Tran Khac, V. (2024). Suivi environnemental des eaux du lac du Bourget pour l'annee 2023. INRAE. https://hal.science/hal-04682522

[jfb70541-bib-0041] Kipling, C. (1976). Year class strengths of perch and pike in Windermere (pp. 68–75). Freshwater Biological Association. http://hdl.handle.net/1834/22681

[jfb70541-bib-0042] Kokkonen, E. , Heikinheimo, O. , Pekcan‐Hekim, Z. , & Vainikka, A. (2019). Effects of water temperature and pikeperch (*Sander lucioperca*) abundance on the stock–recruitment relationship of Eurasian perch (*Perca fluviatilis*) in the northern Baltic Sea. Hydrobiologia, 841(1), 79–94. 10.1007/s10750-019-04008-z

[jfb70541-bib-0043] Laurel, B. J. , Hunsicker, M. E. , Ciannelli, L. , Hurst, T. P. , Duffy‐Anderson, J. , O'Malley, R. , & Behrenfeld, M. (2021). Regional warming exacerbates match/mismatch vulnerability for cod larvae in Alaska. Progress in Oceanography, 193, 102555. 10.1016/j.pocean.2021.102555

[jfb70541-bib-0044] Linløkken, A. N. (2023). Temperature effects on recruitment and individual growth of two antagonistic fish species, perch *Perca fluviatilis* and roach *Rutilus rutilus*, from a climate change perspective. Fishes, 8(6), 295. 10.3390/fishes8060295

[jfb70541-bib-0045] Marcek, B. J. , Farmer, T. M. , Marschall, E. A. , Petris, G. , & Ludsin, S. A. (2021). Ecosystem change as a driver of fish recruitment dynamics: A case study of two Lake Erie yellow perch populations. Freshwater Biology, 66(6), 1149–1168. 10.1111/fwb.13707

[jfb70541-bib-0046] Masson, S. , Angeli, N. , Guillard, J. , & Pinel‐Alloul, B. (2001). Diel vertical and horizontal distribution of crustacean zooplankton and young of the year fish in a sub‐alpine lake: An approach based on high frequency sampling. Journal of Plankton Research, 23(10), 1041–1060.

[jfb70541-bib-0047] Miller, T. J. , Crowder, L. B. , Rice, J. A. , & Marschall, E. A. (1988). Larval size and recruitment mechanisms in fishes: Toward a conceptual framework. Canadian Journal of Fisheries and Aquatic Sciences, 45(9), 9. 10.1139/f88-197

[jfb70541-bib-0048] Mouget, A. , Goulon, C. , Axenrot, T. , Balk, H. , Lebourges‐Dhaussy, A. , Godlewska, M. , & Guillard, J. (2019). Including 38 kHz in the standardization protocol for hydroacoustic fish surveys in temperate lakes. Remote Sensing in Ecology and Conservation, 5(4), 4. 10.1002/rse2.112

[jfb70541-bib-0049] Neuheimer, A. B. , & Taggart, C. T. (2007). The growing degree‐day and fish size‐at‐age: The overlooked metric. Canadian Journal of Fisheries and Aquatic Sciences, 64(2), 375–385. 10.1139/f07-003

[jfb70541-bib-0050] Newsome, G. E. , & Aalto, S. K. (1987). An egg‐mass census method for tracking fluctuations in yellow perch (*Perca flavescens*) populations. Canadian Journal of Fisheries and Aquatic Sciences, 44(6), 6. 10.1139/f87-145

[jfb70541-bib-0051] Ning, N. , Barlow, C. , Baumgartner, L. J. , Bretzel, J. B. , Doyle, K. E. , Duffy, D. , Price, A. , & Vu, A. V. (2025). A global review of the biology and ecology of the European perch, *Perca fluviatilis* . Reviews in Fish Biology and Fisheries, 35(2), 587–618. 10.1007/s11160-025-09924-z

[jfb70541-bib-0052] Ojanguren, A. F. , & Braña, F. (2003). Thermal dependence of embryonic growth and development in brown trout. Journal of Fish Biology, 62(3), 580–590. 10.1046/j.1095-8649.2003.00049.x

[jfb70541-bib-0053] Olin, M. , Malinen, T. , & Ruuhijärvi, J. (2009). Gillnet catch in estimating the density and structure of fish community. Fisheries Research, 96(1), 88–94. 10.1016/j.fishres.2008.09.007

[jfb70541-bib-0054] Peck, M. A. , Huebert, K. B. , & Llopiz, J. K. (2012). Chapter 3—Intrinsic and extrinsic factors driving match–mismatch dynamics during the early life history of marine fishes. In G. Woodward , U. Jacob , & E. J. O'Gorman (Eds.), Advances in ecological research (Vol. 47, pp. 177–302). Academic Press. 10.1016/B978-0-12-398315-2.00003-X

[jfb70541-bib-0055] Pedersen, E. J. , Miller, D. L. , Simpson, G. L. , & Ross, N. (2019). Hierarchical generalized additive models in ecology: An introduction with mgcv. PeerJ, 7, e6876. 10.7717/peerj.6876 PMC6542350 31179172

[jfb70541-bib-0056] Perga, M.‐E. , Bec, A. , & Anneville, O. (2009). Origins of carbon sustaining the growth of whitefish *Coregonus lavaretus* early larval stages in Lake Annecy: Insights from fatty‐acid biomarkers. Journal of Fish Biology, 74(1), 1. 10.1111/j.1095-8649.2008.02105.x 20735521

[jfb70541-bib-0057] Perrier, C. , Molinero, J. C. , Gerdeaux, D. , & Anneville, O. (2012). Effects of temperature and food supply on the growth of whitefish *Coregonus lavaretus* larvae in an oligotrophic peri‐alpine lake. Journal of Fish Biology, 81(5), 1501–1513. 10.1111/j.1095-8649.2012.03393.x 23020558

[jfb70541-bib-0058] Pitlo, J. (2002). Effects of environmental factors on walleye and sauger recruitment in pool 13, upper Mississippi River. North American Journal of Fisheries Management, 22(3), 3. 10.1577/1548-8675(2002)022<1021:EOEFOW>2.0.CO;2

[jfb70541-bib-0059] Pollom, R. A. , & Rose, G. A. (2016). A global review of the spatial, taxonomic, and temporal scope of freshwater fisheries hydroacoustics research. Environmental Reviews, 24(3), 3.

[jfb70541-bib-0060] Pope, K. L. , Willis, D. W. , & Lucchesi, D. O. (1996). Differential relations of age‐0 black crappie and yellow perch to climatological variables in a natural lake. Journal of Freshwater Ecology, 11(3), 345–350. 10.1080/02705060.1996.9664457

[jfb70541-bib-0061] Power, M. , & Van Den Heuvel, M. R. (1999). Age‐0 yellow perch growth and its relationship to temperature. Transactions of the American Fisheries Society, 128(4), 687–700. 10.1577/1548-8659(1999)128<0687:AYPGAI>2.0.CO;2

[jfb70541-bib-0062] Prats, J. , & Danis, P.‐A. (2019). An epilimnion and hypolimnion temperature model based on air temperature and lake characteristics. Knowledge & Management of Aquatic Ecosystems, 420, 8. 10.1051/kmae/2019001

[jfb70541-bib-0082] R Core Team . (2025). R: A Language and Environment for Statistical Computing. R Foundation for Statistical Computing. https://www.R-project.org/

[jfb70541-bib-0063] Rautureau, C. , Goulon, C. , & Guillard, J. (2022). In situ TS detections using two generations of echo‐sounder, EK60 and EK80: The continuity of fishery acoustic data in lakes. Fisheries Research, 249, 106237. 10.1016/j.fishres.2022.106237

[jfb70541-bib-0064] Rimet, F. , Anneville, O. , Barbet, D. , Chardon, C. , Crépin, L. , Domaizon, I. , Dorioz, J.‐M. , Espinat, L. , Frossard, V. , Guillard, J. , Goulon, C. , Hamelet, V. , Hustache, J.‐C. , Jacquet, S. , Lainé, L. , Montuelle, B. , Perney, P. , Quetin, P. , Rasconi, S. , … Monet, G. (2020). The observatory on LAkes (OLA) database: Sixty years of environmental data accessible to public. Journal of Limnology, 79(2), 165–179. 10.4081/jlimnol.2020.1944

[jfb70541-bib-0065] Romare, P. (2000). Growth of larval and juvenile perch: The importance of diet and fish density. Journal of Fish Biology, 56(4), 4. 10.1111/j.1095-8649.2000.tb00878.x

[jfb70541-bib-0066] Schindler, D. W. (1978). Factors regulating phytoplankton production and standing crop in the world's freshwaters. Limnology and Oceanography, 23(3), 3.

[jfb70541-bib-0067] Schmitz, A. M. , & Sepulveda Villet, O. J. (2021). Short term temperature fluctuations affect embryonic and larval development of yellow perch (*Perca flavescens*). Journal of Aquaculture & Marine Biology, 10(Issue 4), 168–176. 10.15406/jamb.2021.10.00318

[jfb70541-bib-0068] Sharaf, N. , Prats, J. , Reynaud, N. , Tormos, T. , Bruel, R. , Peroux, T. , & Danis, P.‐A. (2023). A long‐term dataset of simulated epilimnion and hypolimnion temperatures in 401 French lakes (1959–2020). Earth System Science Data, 15(12), 5631–5650. 10.5194/essd-15-5631-2023

[jfb70541-bib-0069] Simpson, G. L. (2024). Gratia: An R package for exploring generalized additive models. Journal of Open Source Software, 9(104), 6962. 10.21105/joss.06962

[jfb70541-bib-0070] Simpson, G. L. , & Singmann, H. (2022). gratia: Graceful ‘ggplot’‐Based Graphics and Other Functions for GAMs Fitted Using ‘mgcv’ . (Version 0.7.3) [Computer software]. https://CRAN.R-project.org/package=gratia

[jfb70541-bib-0071] Sotton, B. , Anneville, O. , Cadel‐Six, S. , Domaizon, I. , Krys, S. , & Guillard, J. (2011). Spatial match between *Planktothrix rubescens* and whitefish in a mesotrophic peri‐alpine lake: Evidence of toxins accumulation. Harmful Algae, 10(6), 6.

[jfb70541-bib-0072] Takasuka, A. , Nishikawa, H. , Furuichi, S. , & Yukami, R. (2021). Revisiting sardine recruitment hypotheses: Egg‐production‐based survival index improves understanding of recruitment mechanisms of fish under climate variability. Fish and Fisheries, 22(5), 974–986. 10.1111/faf.12564

[jfb70541-bib-0073] Thorpe, J. E. (2011). Morphology, physiology, behavior, and ecology of *Perca fluviatilis* L. and *Perca flavescens* Mitchill. Journal of the Fisheries Board of Canada (Ottawa, Canada), 34(10), 1504–1514. 10.1139/f77-215

[jfb70541-bib-0074] Van De Hey, J. A. , Kaemingk, M. A. , Jansen, A. C. , Graeb, B. D. S. , Dembkowski, D. J. , & Willis, D. W. (2013). Effects of simulated cold fronts on the survival and behaviour of yellow perch *Perca flavescens* yolk‐sac fry. Journal of Applied Ichthyology, 29(2), 364–367. 10.1111/jai.12115

[jfb70541-bib-0075] Vasseur, D. A. , DeLong, J. P. , Gilbert, B. , Greig, H. S. , Harley, C. D. G. , McCann, K. S. , Savage, V. , Tunney, T. D. , & O'Connor, M. I. (2014). Increased temperature variation poses a greater risk to species than climate warming. Proceedings of the Royal Society B: Biological Sciences, 281(1779), 20132612. 10.1098/rspb.2013.2612 PMC3924069 24478296

[jfb70541-bib-0076] Vlavonou, R. S. (1996). Elevage expérimental de la perche *Perca fluviatilis* L.: Développement larvaire et croissance. [Phdthesis, Université Paul Verlaine‐Metz]. https://hal.univ-lorraine.fr/tel-01777177

[jfb70541-bib-0077] Wang, N. (1994). Food and feeding of young perch (*Perca fluviatilis* L.) in Lake Constance. SIL Proceedings, 25(4), 4.

[jfb70541-bib-0078] Wang, N. , & Eckmann, R. (1994). Effects of temperature and food density on egg development, larval survival and growth of perch (Perca fluviatilis L.). Aquaculture, 122(4), 4.

[jfb70541-bib-0079] Ward, M. J. , Anderson, M. R. , Fisher, S. J. , Isermann, D. A. , Phelps, Q. E. , & Willis, D. W. (2004). Relations between climatological variables and larval yellow perch abundance in eastern south Dakota glacial lakes. Journal of Freshwater Ecology, 19(2), 2. 10.1080/02705060.2004.9664534

[jfb70541-bib-0080] Wood, S. (2022). mgcv: Mixed GAM Computation Vehicle with Automatic Smoothness Estimation . (Version 1.8‐40) [Computer software]. https://CRAN.R-project.org/package=mgcv

[jfb70541-bib-0081] Yule, D. L. , Evrard, L. M. , Cachera, S. , Colon, M. , & Guillard, J. (2013). Comparing two fish sampling standards over time: Largely congruent results but with caveats. Freshwater Biology, 58(10), 2074–2088.

